# Investigation of the Optimum Baseline Blood Pressure for Spinal Anesthesia to Guide Vasopressor Management for Elective Cesarean Delivery: A Case-Control Design

**DOI:** 10.7759/cureus.45380

**Published:** 2023-09-16

**Authors:** Vesela P Kovacheva, William Armero, Guohai Zhou, David Bishop, Robert Dyer, Brendan Carvalho

**Affiliations:** 1 Anesthesiology, Brigham and Women's Hospital, Harvard Medical School, Boston, USA; 2 Anesthesiology, Perioperative and Pain Medicine, Brigham and Women’s Hospital, Boston, USA; 3 Center for Clinical Investigation, Brigham and Women’s Hospital, Boston, USA; 4 Anaesthetics, Critical Care and Pain Management, University of KwaZulu-Natal, Scottsville, ZAF; 5 Anaesthesia and Perioperative Medicine, Groote Schuur Hospital Observatory, University of Cape Town, Cape Town, ZAF; 6 Anesthesiology, Stanford University, Stanford, USA

**Keywords:** phenylephrine, mean arterial pressure, spinal hypotension, blood pressure, intraoperative, cesarean, spinal anesthesia, prophylactic vasopressor administration

## Abstract

Background: Current guidelines recommend prophylactic vasopressor administration during spinal anesthesia for cesarean delivery to maintain intraoperative blood pressure above 90% of the baseline value. We sought to determine the optimum baseline mean arterial pressure (MAP) reading to guide the management of spinal hypotension.

Methods: We performed a secondary analysis of data collected from normotensive patients presenting for elective cesarean delivery in a tertiary care institution from October 2018 to August 2020. We compared the magnitude of hypotension in patients who reported nausea versus those who did not, using a case-control design. Baseline MAPs at last office visit, morning of surgery, or operating room (pre-spinal) were determined. We calculated the duration and degree of hypotension using the area under the curve (AUC) when the MAP of the respective patient was below 90% of each baseline.

Results: The patients who experienced nausea (n=45) had longer and more profound periods of hypotension than those who did not develop nausea (n=240). A comparison of AUC using MAP baseline at the last office visit or on the morning of surgery showed a statistically significant between-group difference, P=0.02, and P=0.005, respectively, and no significant between-group difference when 90% of the MAP baseline in the operating room was used.

Conclusions: Patients had the highest preoperative MAP in the operating room and the AUC was similar for those with and without nausea when the pre-spinal MAP baseline was used. Therefore, maintaining higher intraoperative blood pressure using individual pre-spinal MAP as baseline should reduce intraoperative maternal nausea.

## Introduction

Spinal hypotension during cesarean delivery occurs in up to 80% of patients [1]. Hypotension in this setting is associated with side effects for the mother (nausea, vomiting, and cardiovascular collapse) and neonate (decreased uteroplacental perfusion and acidosis) [2,3]. Nausea is a leading complication that expectant mothers would like to avoid [4]. Maternal and neonatal outcomes are likely influenced by the blood pressure at which vasopressor therapy is initiated. Current guidelines recommend prophylactic administration of a vasopressor, most commonly phenylephrine, and maintenance of the intraoperative blood pressure above 90% of the baseline value [1]. A randomized controlled trial demonstrated that maintaining intraoperative blood pressure at baseline values using crystalloid co-load and phenylephrine infusion was associated with the lowest incidence of maternal nausea and vomiting, and optimal neonatal acid-base status [5].

Baseline blood pressure is commonly defined as a single or average measurement obtained in the operating room before the administration of spinal anesthesia [6]. In most pregnant patients, the blood pressure on the day of surgery and in the operating room is significantly higher than the resting blood pressure measured during the office visit or at home [7,8]. Therefore, selecting the office blood pressure or the blood pressure in the ward on the morning of surgery as the baseline to maintain during cesarean delivery may result in inappropriately low doses of vasopressor and a higher incidence of nausea.

We sought to determine the optimal baseline value to guide blood pressure control in patients presenting for elective cesarean delivery under spinal anesthesia. Specifically, this retrospective analysis explored different targets based on the blood pressure measured (i) at the last predelivery office visit, (ii) between 7 am and 12 pm on the day of the surgery, and (iii) in the operating room before the administration of spinal anesthesia, to determine which value was associated with the lowest incidence of nausea or vomiting. Our primary outcome was the presence or absence of nausea and vomiting associated with maintaining the mean arterial blood pressure (MAP) within 90% of one of the following three hemodynamic targets: baseline MAP at the last office visit, morning of surgery, or operating room (pre-spinal).

## Materials and methods

Following IRB approval (#2017P002304), with a waiver of patient consent, we performed a secondary post-hoc analysis of data collected in a previous investigation, to determine the optimal baseline blood pressure for the management of spinal hypotension during cesarean delivery. The original study was designed to collect precise data with the goal of creating an algorithm for the prediction of systolic blood pressure and the determination of the phenylephrine dose required to maintain hemodynamic stability after spinal anesthesia for cesarean delivery [9].

Original study design

The original study collected data from women who had scheduled cesarean delivery at the Brigham and Women’s Hospital from October 2017 to August 2020 [9]. Precise electronic records were made of the timing and dose of all intraoperative medications, the occurrence of nausea, vomiting, flushing, and lightheadedness, from the time of administration of the neuraxial anesthesia until delivery of the neonate. The patients did not receive any specific instructions or prompts regarding reporting the occurrence of nausea, vomiting, flushing, and lightheadedness; if any symptoms were spontaneously reported or witnessed, these were recorded by an independent observer.

All data were obtained from the electronic health record. American Society of Anesthesiologists (ASA) class 2 and 3 patients were included who had cesarean delivery under spinal or combined spinal-epidural anesthesia; the latter mode of anesthesia was selected only if the epidural component had not been activated during the data collection period. All patients with cardiovascular disease (for example, hypertensive disorders of pregnancy), patients taking cardiovascular medications, those with inaccurate records, or patients who received vasopressors before the spinal anesthesia placement were excluded. Preoperative data collected included patient age, height, weight, gestational age, gravidity, parity, spontaneous rupture of membranes, contractions, and comorbidities (diabetes, anxiety, depression). Intraoperative data collected consisted of medications (bupivacaine, phenylephrine, and ephedrine), side effects (nausea, vomiting, lightheadedness), vital signs (heart rate and blood pressure), and Apgar scores of the neonate. The intraoperative data were collected for the period of five minutes before the spinal injection and continued for 20 minutes after injection or until delivery of the neonate, at intervals of one minute. When available on the weekdays, a research assistant was present in the operating room during the case, who acted as a scribe and assisted the clinical team in precisely documenting in the electronic record the administered medications, and the presence or absence of any intraoperative events. Should the patient spontaneously complain of nausea and/or vomiting, this was recorded; otherwise, the absence of nausea was documented.

The clinical care of the patients followed standard institutional care. The premedication included oral sodium citrate 30 ml, metoclopramide 10-20 mg, and the preoperative intravenous administration of 500 ml lactated Ringer’s solution over 20 minutes. Upon arrival in the operating room, the patients were positioned in the sitting position, and standard monitors were applied, including noninvasive blood pressure, continuous electrocardiogram, and pulse oximetry using GE Carescape B650 monitor (GE Healthcare, USA). The noninvasive blood pressure was measured in the sitting position until spinal anesthesia was complete and thereafter in the supine position, with the goal of measurement at one-minute intervals. Spinal anesthesia was induced using 1.4-2.0 ml 0.75% hyperbaric bupivacaine with 100-200 µg morphine and 10-15 µg fentanyl. The patient was immediately positioned supine with standard left lateral tilt. At the time of the spinal injection, a rapid bolus of 500-1,000 ml lactated Ringer’s solution was administered (co-load) in addition to the preoperative hydration. Prophylactic ondansetron 4 mg was administered intravenously after induction of spinal anesthesia. Prophylactic intravenous phenylephrine infusion at a rate of 40-60 µg/min was used. Vasopressor management was directed at maintaining the systolic blood pressure above 90 mmHg (target based on the institutional protocol during the study period) using by titration of the intravenous phenylephrine infusion, supplemented by bolus phenylephrine 80-160 µg or, if the heart rate was lower than 60 beats per minute, a bolus of 5-10 mg ephedrine. 

Current secondary analysis study design

For purposes of this study, we selected all records in which a research assistant was present up to the delivery time, and there was documentation of the presence or absence of nausea and/or vomiting. Assuming 45% incidence of hypotension among patients with nausea, and 20% incidence of hypotension among patients without nausea, 80% power (5% type I error or P<0.05 considered as statistical significance) required 37 patients with intraoperative nausea and 148 patients without nausea [10].

In addition to the data collected above in the original study, we obtained from the medical record the blood pressure and heart rate recorded at the last outpatient visit and on the day of the surgery. To establish which baseline blood pressure reading would have been associated with the least nausea and vomiting, we defined the MAP taken at the last office visit as MAP_LV, the MAP in the morning of surgery as MAP_AM, and the MAP averaged for the last five minutes before the spinal administration as MAP_pre-spinal. These readings were obtained in the sitting position using a noninvasive blood pressure cuff. We also defined the BP target range as MAP 90% -110% of the baseline and investigated three different scenarios using MAP_LV, MAP_AM, and MAP_pre-spinal anesthesia as the baseline.

Statistical analysis

**Figure 1:**
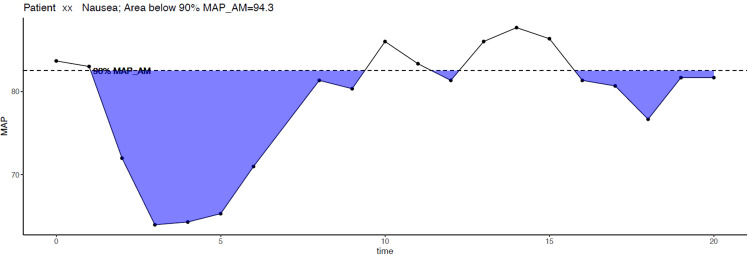
Calculation of the AUC Nausea; Area below 90% MAP_AM=94.3.  For this patient, we determined the 90% MAP_AM and calculated the extent of the MAP decrease and time spent below that goal. MAP_AM: Mean arterial blood pressure in the morning of surgery; AUC: Area under the curve

For analysis purposes, the study population was divided into two groups: patients who reported nausea and/or vomiting (nausea) and those who did not (non-nausea). Missing blood pressure and heart rate values were linearly interpolated or extended. Continuous variables were not normally distributed and expressed as median (interquartile range), and categorical variables were defined as number/total number (%). We calculated the time and deviation from the target MAP values using the area under the curve (AUC) trapezoidal rule [11,12]. We selected the AUC variable (Figure 1) since it can accommodate both changes from the baseline MAP target and the time interval during which there was hypotension. We used MAP_LV, MAP_AM, and MAP_pre-spinal as a baseline and calculated the AUC when the MAP of the respective patient was below 90% of each hypothetical target. Between-group comparisons of continuous variables were performed using the Wilcoxon test, and using the Fisher’s exact test for categorical variables. Vasopressor use was expressed in phenylephrine equivalents, which was the total amount of phenylephrine plus ephedrine, converted at a ratio of ephedrine 1 mg = phenylephrine 12.5 µg [13]. We calculated wobble as a measure of inter-subject variability [14]. We defined the degree of hypotension as the difference between the baseline MAP and the lowest post-spinal MAP value; and duration of hypotension as the time spent with MAP below the baseline. Statistical significance was defined as P<0.05. Data analysis was performed using R version 4.1.0 (www.R-project.org). 

## Results

There were 285 patients who met the inclusion criteria, all of whom were included in the present study. The patient characteristics are presented in Table 1. There were 45/285 patients (15.8%) who developed nausea and one patient who developed both nausea and vomiting. There were no statistically significant differences between the women who did or did not develop intraoperative nausea in terms of demographic characteristics, presence of early labor (either spontaneous rupture of membranes or regular contractions), and comorbidities such as diabetes and depression.

**Table 1:** Patient characteristics Median (interquartile range) for continuous variables; n (%) for categorical variables; P-values for continuous variables based on Wilcoxon test and for categorical variables based on Fisher’s exact test.

	Nausea (n=45)	No nausea (n=240)	P-value
Age (y)	35.0 (32.0 - 38.0)	35.0 (32.0 - 37.0)	0.431
BMI (kg/m^2^)	31.0 (27.1 - 34.5)	31.1 (27.2 - 34.7)	0.803
Gravidity	2.0 (2.0 - 3.0)	2.0 (2.0 - 3.0)	0.701
Parity	1.0 (0.0 - 1.0)	1.0 (0.0 - 1.0)	0.945
Gestational age at delivery (weeks)	38.7 (37.7 - 39.3)	39.0 (37.9 - 39.4)	0.264
Contractions	8 (17.8%)	30 (12.5%)	0.609
Spontaneous rupture of membranes	3 (6.7%)	15 (6.2%)	1
Gestational diabetes	2 (4.4%)	29 (12.1%)	0.191
Diabetes type 2	1 (2.2%)	1 (0.4%)	0.291
Insulin use	2 (4.4%)	12 (5.0%)	1
Depression/anxiety	12 (26.7%)	67 (27.9%)	1
Apgar score at 1 min	8 (8-8)	8 (8-8)	0.310
Apgar score at 5 min	9 (8-9)	9 (8-9)	1.000

**Figure 2:**
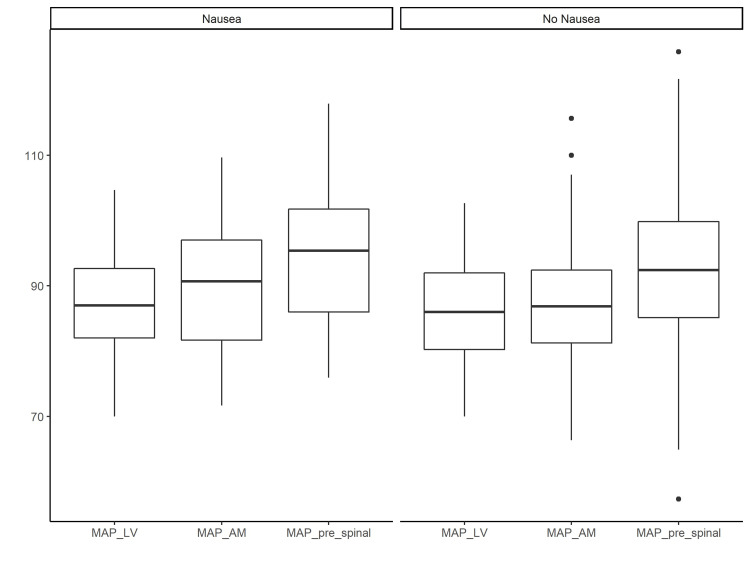
Comparison of the MAP at the last office visit, the morning of surgery, and in the operating room before spinal anesthesia MAP_LV: Mean arterial blood pressure taken at the last office visit; MAP_AM: MAP in the morning of surgery; MAP_pre-spinal: MAP averaged for the last five minutes before the spinal administration

Table 2 shows between-group differences in heart rate, MAP, wobble, and phenylephrine equivalents. The baseline heart rate was similar in both groups. There were no significant differences in the MAP_LV, MAP_AM, and MAP_pre-spinal baseline target values in the nausea and non-nausea groups. Intraoperatively, the group that experienced nausea had lower MAP and received more ephedrine, phenylephrine and phenylephrine equivalents compared with the control group (P<0.05). Overall, the between-group comparison of MAP measured at MAP_LV, MAP_AM, and MAP_pre-spinal demonstrated a significant effect of time, P<0.0001, and group, P=0.02 (Figure 2).

**Table 2:** Between-group differences in heart rate, MAP and phenylephrine equivalents Values are median (interquartile range). MAP_LV: Mean arterial blood pressure taken at the last office visit; MAP_AM: MAP in the morning of surgery; MAP_pre-spinal: MAP averaged for the last five minutes before the spinal administration

	Nausea (n=45)	No nausea (n=240)	P-value
Heart rate in the operating room before the spinal, bpm	94.8 (85.9 - 105.6)	90.0 (82.5 - 98.9)	0.068
Lowest heart rate after the spinal, bpm	60.0 (54.0 - 69.0)	63.0 (57.0 - 71.0)	0.125
MAP_pre-spinal, mmHg	95.4 (86.0 - 101.8)	92.4 (85.1 - 99.8)	0.297
MAP_AM, mmHg	90.7 (81.7 - 97.0)	86.8 (81.2 - 92.4)	0.125
MAP_LV, mmHg	87.0 (82.0 - 92.7)	86.0 (80.2 - 92.0)	0.198
Wobble (%) of MAP, MAP_pre-spinal as baseline	6.6 (4.4 - 11.0)	7.2 (4.1 - 15.4)	0.758
Wobble (%) of MAP, MAP_AM as baseline	5.8 (3.8 - 9.1)	4.5 (3.2 - 7.2)	0.055
Wobble (%) of MAP, MAP_LV as baseline	5.7 (4.0 - 10.0)	4.5 (3.2 - 7.5)	0.055
Lowest MAP after the spinal, mmHg	64.0 (58.7 - 73.0)	69.7 (64.3 - 73.7)	0.005
Average MAP over 20 min after the spinal, mmHg	79.6 (76.4 - 83.8)	79.0 (75.6 - 83.6)	0.765
Total ephedrine for the first 20 min, mg	0 (0-10)	0 (0-0)	0.004
Total phenylephrine equivalents for the first 20 min, µg	1100.0 (800.0 - 1426.0)	840.0 (660.0 - 1080.4)	0.001

**Figure 3:**
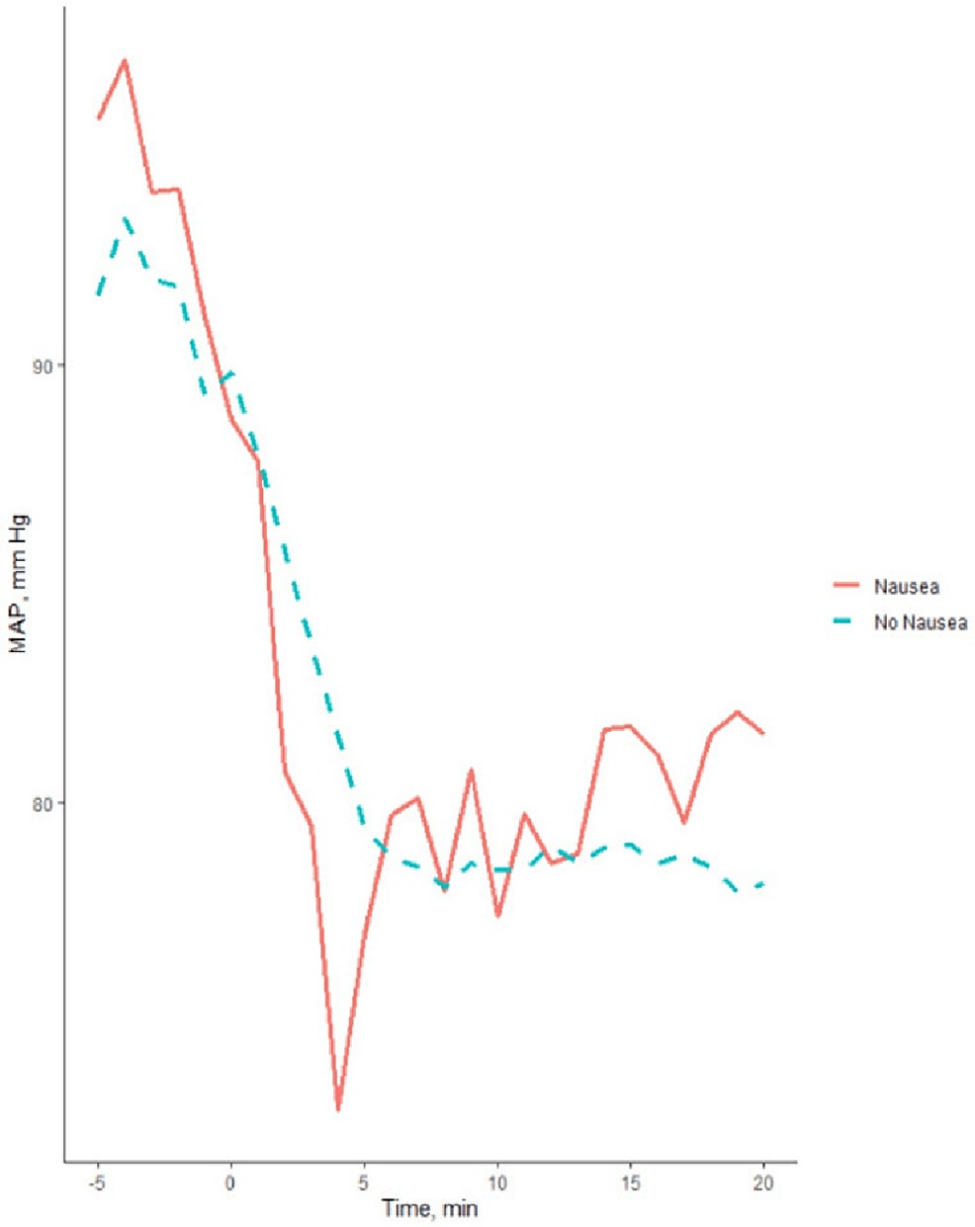
Intraoperative arterial blood pressure course MAP time course in nausea and non-nausea groups averaged for all patients in each group; spinal anesthesia is administered at time 0. MAP: Mean arterial blood pressure

Figure 3 shows the mean MAP values at each time point in the nausea (n=45) versus non-nausea (n=240) groups. A comparison of AUC using MAP_LV and MAP_AM baseline showed a statistically significant difference between women developing and not developing nausea (P=0.02 and P=0.005, respectively) (Table 3). There was no significant between-group difference in AUC when MAP_pre-spinal baseline was used. Using a target MAP of 90% of MAP_pre-spinal as a goal for intraoperative management, 89% of the patients in the nausea group and 84% of the patients in the non-nausea group did not reach this goal. We further investigated which components of hypotension contributed to the outcome. The average duration of hypotension, defined as time with MAP below baseline, was 16 minutes, and there was no difference between the groups, P=0.7. The patients with nausea experienced a significant degree of hypotension, defined as the difference between MAP_pre-spinal and the lowest post-spinal MAP, on average 30 mmHg, compared with the group without nausea, 24 mmHg (P=0.001). Using MAP_prespinal as the baseline, there were 4 (8.89%) patients with normal blood pressure (90-110% of baseline) who developed nausea; 41 (91.1%) patients in the nausea group had low blood pressure.

**Table 3:** A between-group comparison of the AUC < 90% of baseline MAP in the operating room before spinal administration, on the morning of surgery, and at the last office visit A between-group comparison of the AUC < 90% of baseline MAP in the operating room before spinal administration, on the morning of surgery, and at the last office visit AUC: Area under the curve; MAP_LV: Mean arterial blood pressure taken at the last office visit; MAP_AM: MAP in the morning of surgery; MAP_pre-spinal: MAP averaged for the last five minutes before the spinal administration

	Nausea (n=45)	Non-nausea (n=240)	P-value
AUC < 90% MAP_pre-spinal	112.5 (50.0 - 228.5)	86.4 (14.5 - 161.7)	0.094
AUC < 90% MAP_AM	69.4 (19.9 - 188.6)	33.7 (2.8 - 84.6)	0.005
AUC < 90% MAP_LV	51.6 (20.1 - 104.4)	26.2 (0.7 - 69.9)	0.016

## Discussion

This study investigated the perioperative blood pressure time course in pregnant patients undergoing elective cesarean delivery. We explored three hypothetical hemodynamic targets based on the blood pressure measurements obtained at different times before the administration of spinal anesthesia, with the goal of decreasing the risk of intraoperative nausea and vomiting: MAP_LV, MAP_AM, and MAP_pre-spinal. We demonstrated that patients had the highest preoperative MAP in the operating room before the administration of spinal anesthesia (MAP_pre-spinal). The duration and degree of hypotension was estimated using the AUC when the MAP of the respective patient was below 90% of each baseline. Patients who experienced nausea had longer and more profound periods of hypotension compared to the controls, as evidenced by the lower MAP after the administration of spinal anesthesia. Selecting a target for blood pressure during spinal anesthesia within 90% of the blood pressure measured at the last outpatient visit (MAP_LV) or on the morning of surgery (MAP_AM) resulted in more episodes of hypotension in the nausea group than in the group without nausea, i.e., significant between-group differences between the AUCs for patients with and without nausea. Therefore, selecting either of these low targets would result in overall lower blood pressure management and a higher incidence of nausea. In contrast, there was no significant between-group difference for AUC when selecting within 90% of the pre-spinal MAP values (MAP_pre-spinal) as a hemodynamic goal. Thus, higher blood pressures would be maintained using MAP_pre-spinal baseline in both groups, which would result in the lowest incidence of hypotension and, respectively, nausea. These results suggest that MAP pre-spinal should be selected as the optimum baseline blood pressure for managing spinal hypotension to minimize nausea and vomiting during cesarean delivery. 

Our results agree with previous findings that blood pressure measured in the operating room immediately before administering anesthesia is higher than at the last office visit and on the morning of surgery [6-8,15]. Large cohort studies of non-obstetric patients presenting for surgery demonstrate that pre-induction, compared to preoperative, MAP is higher on average by 3-17 mm Hg, with a wide inter-individual variation [7,15-17]. Similarly, in obstetric patients presenting for elective cesarean delivery, the blood pressure is 5 mmHg higher on the morning of surgery than at the last office visit, and 15 mmHg higher pre-induction than on the morning of surgery, with a wide inter-individual variation [8]. This blood pressure elevation is likely related to stress in anticipation of induction of anesthesia and surgery.

Hypotension is the most common cause of intraoperative nausea in patients presenting for cesarean delivery under spinal anesthesia. The incidence of nausea in our cohort was 15.8%, similar to other studies in which patients have self-reported their symptoms [10]. We believe that our results have a low risk of bias, as we used a research assistant to ensure precise documentation, and, on the days when he was available, all eligible patients were recorded and included in this study. Due to our study design and our clinical practice relying on patient-reported symptoms and not specifically inquiring about symptoms, it is possible that nausea was under-reported.

Prophylactic vasopressor infusions are recommended to avoid spinal hypotension [1]. Most patients in our study received prophylactic phenylephrine infusion, which is associated with a lower incidence of nausea than rescue bolus dosing [18]. The patients who developed nausea received, on average, 1,110 µg phenylephrine, and 89% of those patients were below the 90% pre-spinal MAP target. Higher doses of vasopressors might have been necessary to reach the defined 90% pre-spinal MAP target. Spinal anesthesia for cesarean delivery is typically associated with arteriolar dilatation, a decrease in systemic vascular resistance (SVR), hypotension, a well-preserved heart rate, and an increased cardiac output [19]. If preload is adequate, administration of phenylephrine results in restoration of SVR and blood pressure, with a reflex slowing of the heart rate and reduction of the cardiac output to baseline values [20-23]. Excessive doses of phenylephrine would result in a reduction of heart rate and cardiac output to below baseline values, as well as an increase in blood pressure to above the 110% upper limit set in this study. We do not have measures of the cardiac output of the study patients; however, since most patients had intraoperative blood pressures lower than baseline values, we anticipate that cardiac output increases would have been only partially restored to baseline by the phenylephrine infusion at the rates employed.

Our recommendations should lead to the maintenance of higher intraoperative blood pressure than would be the case if the alternative baseline targets were used. In a similar fashion, a randomized trial of 25 parturients per group documented significantly lower rates of nausea when the blood pressure was maintained at 100% of the baseline, compared to 80% and 90% of baseline [5]. The baseline in that study was defined as the blood pressure measurement obtained in the operating room, with the patient in the supine position at rest.

This study was subject to some limitations. Due to the retrospective design, the patients’ blood pressure was maintained at a level deemed appropriate by the clinical team, and confounding variables may not have been excluded. In addition, maternal cardiac output and neonatal cord blood values were not monitored or tested in these elective cases. Missing MAP data in some patients required interpolation. We did not include hypertensive or preeclamptic patients, since blood pressure may be more labile and the response to vasopressors more variable. The nausea was self-reported by the patients, and, therefore, we may have underestimated the incidence. We utilized a research assistant as a scribe to document the patient-reported symptoms precisely. The small sample size may present a risk for selection bias; however, our incidence of nausea was similar to that reported in the literature, suggesting that under- or over-sampled either of the study groups was unlikely. Future larger studies using methods such as continuous cardiac output and blood pressure may generate further insights into the factors associated with nausea and vomiting. We selected only patients presenting for elective cesarean delivery, since obstetric emergencies may be associated with multiple factors such as hemorrhage, patient anxiety, and short anesthesia induction to delivery time, which may confound the results.

## Conclusions

Overall, we demonstrated that patients presenting for elective cesarean delivery had the highest preoperative MAP in the operating room before the administration of spinal anesthesia. Those who experienced nausea had longer and more profound periods of hypotension. There was no difference in AUC in the groups experiencing nausea versus no nausea, when 90% of the MAP baseline in the operating room was used. Based on these results, targeting higher intraoperative blood pressure using the individual preoperative MAP prior to spinal anesthesia as a baseline is preferable, to reduce intraoperative maternal nausea and vomiting.
